# Genetic Profile of Epidermolysis Bullosa Cases in King Abdulaziz Medical City, Riyadh, Saudi Arabia

**DOI:** 10.3389/fgene.2021.753229

**Published:** 2022-02-10

**Authors:** Raghad Alharthi, Muhannad A. Alnahdi, Ahad Alharthi, Seba Almutairi, Sultan Al-Khenaizan, Mohammed A. AlBalwi

**Affiliations:** ^1^ College of Medicine, King Saud Bin Abdulaziz University for Health Sciences, Riyadh, Saudi Arabia; ^2^ Department of Dermatology, King Abdulaziz Medical City, Ministry of National Guard Health Affairs, Riyadh, Saudi Arabia; ^3^ Department of Ophthalmology, Ministry of National Guard Health Affairs, King Abdulaziz Medical City, Riyadh, Saudi Arabia; ^4^ Department of Dermatology, King Fahad University Hospital, Al Khobar, Saudi Arabia; ^5^ Department of Pathology and Laboratory Medicine, Ministry of National Guard Health Affairs, King Abdulaziz Medical City, Riyadh, Saudi Arabia; ^6^ Medical Genomic Research Department, Ministry of National Guard Health Affairs, King Abdullah International Medical Research Center, Riyadh, Saudi Arabia

**Keywords:** epidermolysis bullosa, dystrophic epidermolysis bullosa, junctional epidermolysis bullosa, epidermolysis bullosa simplex, Saudi Arabia

## Abstract

Epidermolysis bullosa (EB) is a rare heterogeneous genetic mechanobullous skin disorder that is characterized by increased skin fragility leading to blistering following minor trauma. EB may be inherited as an autosomal dominant or an autosomal recessive disorder and can be classified into dystrophic EB (DEB), junctional EB (JEB), and EB simplex (EBS). A total of 28 Saudi patients with EB were included in this observational, retrospective chart-review study. A consecutive non-probability sampling technique was used to approach all affected patients. Molecular analysis was done to test the patients’ genomic DNA using a custom-designed AmpliSeq panel of suspected genes. All disease-causing variants were checked against available public databases. Twelve patients (42.9%) were found to have DEB, 6 patients (21.4%) with JEB, and 10 patients (35.7%) with EBS. The molecular genetic results revealed detections of 24 various homozygous genetic variations in the genes associated with EB, of which 14 were novel mutations. The most frequent variations were detected in *COL7A1* in 12 cases (42.9%), followed by *LAMB3* in 5 cases (17.9%), *TGM5* in 4 cases (14.3%), and other genes. Furthermore, the majority (87.5%) of EB cases were confirmed to have homozygous mutations, and few were documented with positive consanguinity history. Only 3 cases (12.5%) were found to be autosomal dominant displaying heterozygous mutations. This is the first study to establish the EB genetic profile in Saudi Arabia where DEB is the most frequent type. A total of 14 novel mutations were identified that had not been previously reported. Consanguineous marriage is clearly recognized in the Saudi population; therefore, we propose a nationwide EB program that would help extend the spectrum of the genetic profile and help in the diagnosis and better understanding of this disease.

## Introduction

Inherited epidermolysis bullosa (EB) is a heterogeneous group of skin disorders characterized by increased skin fragility leading to blister formation following minor trauma (Fine 2010; Mariath et al., 2020). Worldwide, it is estimated that the EB prevalence is about 19.6 per one million of live-born infants (Fine 2016). EB may be inherited as either autosomal dominant or autosomal recessive. This disorder is caused so far by more than 29 gene mutations encoding structural proteins within the skin with functional absence or loss that leads to instability of the micro-architectural connections between the dermis and epidermis, leading to blister formation (Has et al., 2020; Mariath et al., 2020). To date, there are over 30 subtypes of EB recognized, which are classified into four major groups based on clinical or molecular studies: dystrophic EB (DEB), junctional EB (JEB), EB simplex (EBS), and recently Kindler syndrome (Has et al., 2020). Kindler syndrome is a rare type of EB caused by mutations in the *FERMT1* gene and is inherited in an autosomal recessive pattern. Dystrophic EB is caused by mutations in the gene encoding type VII collagen leading to the separation of the sub-basal lamina. DEB is inherited in an autosomal recessive or autosomal dominant pattern. Junctional EB results from mutations in genes encoding either laminin-332 or collagen type XVII, resulting in blister formation within the lamina lucida of the basement membrane. JEB is inherited in an autosomal recessive pattern. EBS results from intra-epidermal separation with mild systemic involvement ascribed to mutations encoding *KRT5* and *KRT14*, resulting in a disturbance of the stability of the keratin filament network. EBS is usually inherited in an autosomal dominant pattern, but in rare cases, it is inherited as autosomal recessive.

In Saudi Arabia (SA), few EB cases were reported in the Eastern Province among dermatology clinic case reviews without detailing their genetic characteristics (Fine 2016). EB research is scarce in the region unlike in other parts of the world, so this study aims to highlight the genetic perspective in Saudi EB patients at a tertiary healthcare center.

The EB patients’ quality of life is highly impacted, as even the mildest form of the disorder leads to blisters and wounds that are quite painful (Abahussein et al., 1993; Tabolli et al., 2009). Potential complications are anemia, vocal cord stenosis, obstructive urethral lesions, and scarring and visual impairment (Abahussein et al., 1993; Fine et al., 2008; Fine and Mellerio 2009a; Fine and Mellerio 2009b). Patients have claimed suffering from physical and psychological restrictions like physical pain, lack of engagement in social activities, and embarrassment owing to their skin appearance (Horn and Tidman 2002a; Fine et al., 2009).

## Materials and methods

### Subjects

We performed an observational and retrospective chart-review study of 28 Saudi’ patients at King Abdulaziz Medical City, a tertiary care hospital in Riyadh, SA. The enrolled patients were diagnosed with EB and skin fragility disorders in the period between 1998 and 2020 and treated at the same center under the divisions of dermatology, general pediatrics, ophthalmology, and dentistry. A consecutive non-probability sampling technique was used to review the files of the patients. All required data were retrieved and gathered from the hospital BestCare system as well as from the database of the molecular pathology and genetics laboratory. Institutional Review Board (IRB) approval was obtained from the ethics committee of King Abdullah International Medical Research Center under RC19/250/R. Data collected from the patients’ files include sociodemographic, clinical, laboratory, and genetic data.

### Genetic analysis

Molecular analysis of these cases was carried out by testing genomic DNA and checking for genetic variations of all exons and exon/intron boundaries using a custom-designed AmpliSeq panel that includes the following genes: *CD151*, *CHST8*, *COL17A1*, *COL7A1*, *CSTA*, *DSG1*, *DSG2*, *DSG3*, *DSG4*, *DSP*, *DST*, *EXPH5*, *FERMT1*, *GRIP1*, *ITGA3*, *ITGA6*, *ITGB4*, *KRT1*, *KRT10*, *KRT14*, *KRT5*, *LAMA3*, *LAMB3*, *LAMC2*, *MMP1*, *NID1*, *PKP1*, *PLEC*, and *TGM5.* All disease-causing variants were checked against the Human Gene Mutation Database (HGMD), ClinVar, the Genome Aggregation Database (gnomAD), and the Exome Aggregation Consortium (ExAC). The in silico tools SIFT (http://sift.jcvi.org/), PolyPhen-2 (http://genetics.bwh.harvard.edu/pph2/), and MutationTaster (http://www.mutationtaster.org were used to predict coding variant effects on protein function. The collected data were entered into Microsoft Excel and analyzed using a simple statistical parameter through IBM Statistical Package for Social Sciences (SPSS) version 24. Numerical variables are presented as mean and standard deviation, and categorical variables are presented as frequencies and percentages.

## Results

The population is represented with a 1.3:1 male-to-female ratio, as male patients were 16 (57%) and female patients were 12 (42.9%). The mean age was 8.9 ± 5.4 years old, the youngest patient was 3 years, and the oldest was 21 years old. Positive consanguinity history was documented in 9 patients, while family history was noted in 6 patients.

**FIGURE 1:**
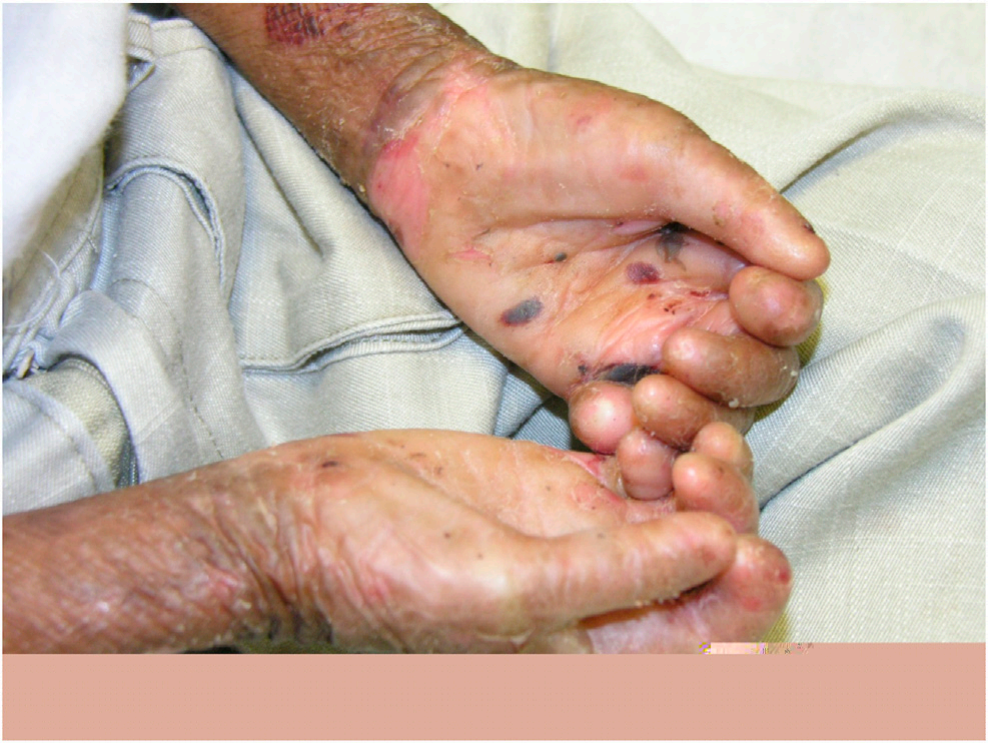
Clinical presentation of dystrophic epidermolysis bullosa.

**FIGURE 2:**
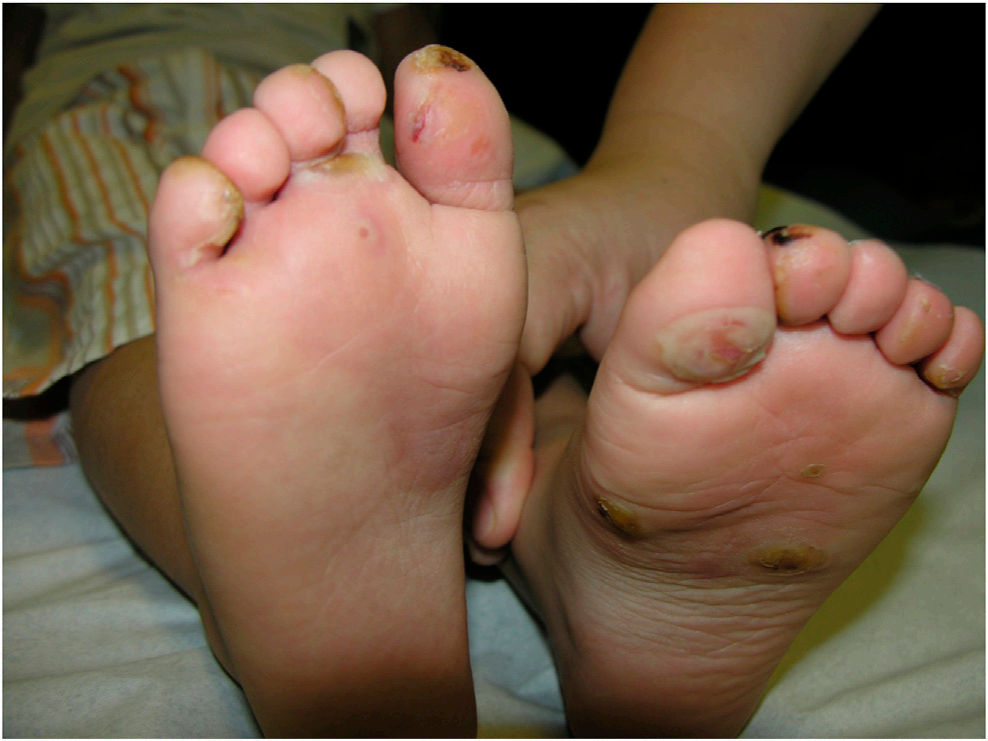
Clinical presentation of epidermolysis bullosa simplex.

**FIGURE 3:**
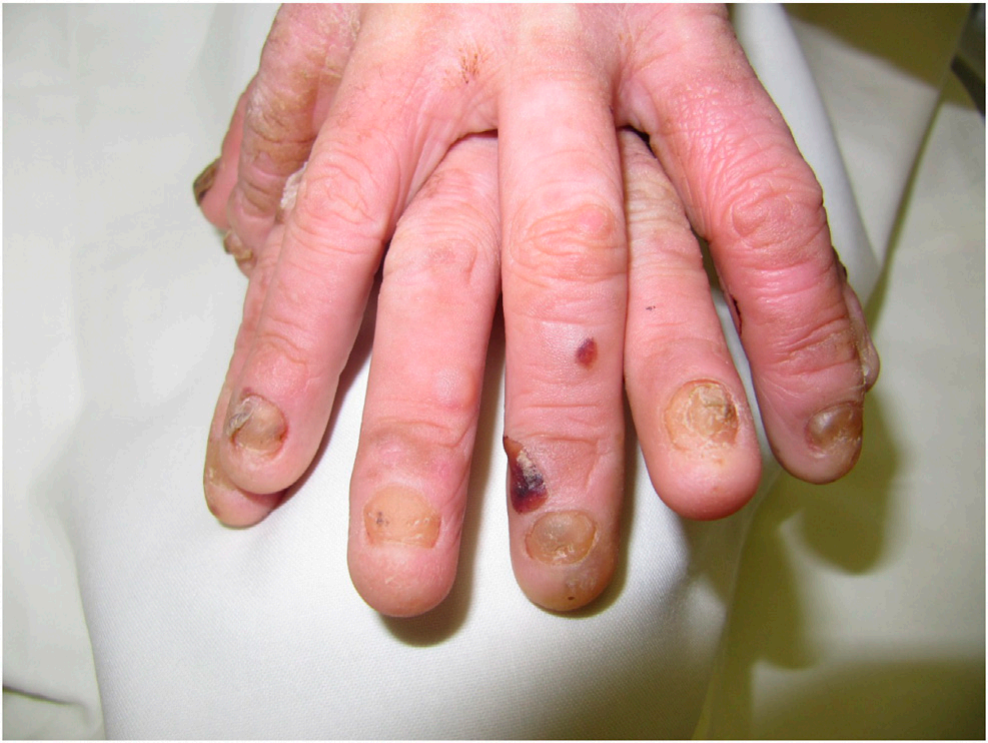
Clinical presentation of junctional epidermolysis bullosa.

Dystrophic EB 12 (42.9%) was the most frequent subtype, followed by EB simplex 10 (35.7%) and junctional EB 6 (21.4%). Phenotypic presentations of each classification are shown in Figures 1–3. The mutations were detected in 7 genes: *COL7A1*, *LAMB3*, *TGM5*, *PLEC*, *DST*, *KRT14*, and *COL17A1*. Mutations implicated with *COL7A1* were the most frequent in which they were found in 12 (42.9%) patients, followed by mutations with *LAMB3* in 5 (17.9%) patients, *TGM5* with 4 (14.3%) patients, *PLEC* with 3 (10.7%) patients, *DST* with 2 (7%) patients, *KRT14* with 1 (3.6%) patient, and *COL17A1* with 1 (3.6%) patient (Table 1).

**TABLE 1:** Frequency of the genes involved in all EB patients.

#	Gene	N=cases	Percentage %
1	COL7A1	12	42.9
2	LAMB3	5	17.9
3	TGM5	4	14.3
4	PLEC	3	10.7
5	DST	2	7.1
6	KRT14	1	3.6
7	COL17A1	1	3.6
Total		28	100

The clinical features of all patients were of the usual phenotype seen in EB patients, namely, mechanobullous fragility and blisters with a wide range of severity according to the genotype. Furthermore, nail deformities, tooth decay, lesions and erosive ulcerations in the oral cavity, and recurrent respiratory and urinary tract infections have been observed. None of patients had any gastrointestinal complications with an exception of one patient who had pyloric atresia. However, we did not detect any unusual other clinical features even in patients with novel mutations.

Genetic analysis of the implicated genes revealed 24 mutations identified among all enrolled cases. Among them, 14 mutations have not been reported to date. Autosomal recessive inheritance prevailed in 25 (89.3%) cases, and only 3 (10.7%) cases were found to be autosomal dominant (Table 2). A total of 4 different mutations were found in more than one patient, and those genes were diagnosed within another member of the same family. A total of 3 cases had the same mutation in *COL7A1* (case nos. 6, 7, and 8), and another mutation of *COL7A1* was found in two cases (case nos. 10, 11). Two cases had the same mutation involved in *LAMB3* (case nos. 17 and 18), and two additional patients had the same gene mutations in *TGM5* (case nos. 26 and 27).

**TABLE 2:** Genes, variants, mutation types, and novelty status per EB classifications.

Case No	Gene	Mutation in cDNA (GenBank ID)	Consequence (protein)	Mutation status	Annotation	*In silico* prediction analysis			Pathogenicity	Reported (dbSNP# or HGMD)
						SIFT	PP	MT		
**Dystrophic epidermolysis bullosa**										
1	*COL7A1*	c.7768G > C (NM_000094.4)	p.Gly2590Arg	Homozygous	Missense	Deleterious	Probably damaging	Disease causing	Pathogenic	Reported rs2043715843
2	*COL7A1*	c.7411C > T (NM_000094.4)	p.Arg2471*	Homozygous	Stop gained	Deleterious	Probably damaging	Disease causing	Pathogenic	Reported rs121912852/CM960412
3	*COL7A1*	c.4520G > T (NM_000094.4)	p.Gly1507val	Homozygous	Missense	Deleterious	Probably damaging	Disease causing	Likely pathogenic	Not reported
4	*COL7A1*	c.4448G > A (NM_000094.4)	p.Gly1483Asp	Homozygous	Missense	Deleterious	Probably damaging	Disease causing	Pathogenic	Reported rs756217590/CM093143
5	*COL7A1*	c.4864G > C (NM_000094.4)	p.Gly1622Arg	Homozygous	Missense	Deleterious	Probably damaging	Disease causing	Pathogenic	Reported CM1618661
6	*COL7A1*	c.4198delG (NM_000094.4)	G1400Vfs*310	Homozygous	Frameshift	Deleterious	Probably damaging	Disease causing	Likely pathogenic	Not reported
7	*COL7A1*	c.4198delG (NM_000094.4)	G1400Vfs*310	Homozygous	Frameshift	Deleterious	Probably damaging	Disease causing	Likely pathogenic	Not reported
8	*COL7A1*	c.4198delG (NM_000094.4)	G1400Vfs*310	Homozygous	Frameshift	Deleterious	Probably damaging	Disease causing	Likely pathogenic	Not reported
9	*COL7A1*	c.611T > G	p.Leu204Ser	Heterozygous Heterozygous	Missense Stop gained	Deleterious	Probably damaging	Disease causing	Likely pathogenic	Reported rs745939385
		Deletion exons 25–52 (NM_000094.4)	p.?							Not reported
10	*COL7A1*	c.1507+1G > C (IVS11+1G > C)	p.?	Homozygous	Splice region	Deleterious	Probably damaging	Disease causing	Likely pathogenic	Reported
		(NM_000094.4)								CS072154
11	*COL7A1*	c.1507+1G > C (IVS11+1G > C)	p.?	Homozygous	Splice region	Deleterious	Probably damaging	Disease causing	Likely pathogenic	Reported
		(NM_000094.4)								CS072154
12	*COL7A1*	c.7442G > A (NM_000094.4)	p.Gly2481Asp	Homozygous	Missense	Deleterious	Probably damaging	Disease causing	Likely pathogenic	Not reported
**Junctional epidermolysis bullosa**										
13	*COL17A1*	c.3922delA (NM_000494.4 )	p.Ser1308Alafs*4	Homozygous	Frameshift	Deleterious	Probably damaging	Disease causing	Likely pathogenic	Not reported
14	*LAMB3*	c.972delA (NM_000228.3)	p.Cys325Serfs*71)	Homozygous	Frameshift	Deleterious	Probably damaging	Disease causing	Likely pathogenic	Not reported
15	*LAMB3*	c.972delA (NM_000228.3)	p.Cys325Serfs*71	Homozygous	Frameshift	Deleterious	Probably damaging	Disease causing	Likely pathogenic	Not reported
16	*LAMB3*	c.1978C > T (NM_000228.3)	p.Arg660*	Homozygous	Stop gained	Deleterious	Probably damaging	Disease causing	Pathogenic	Reported rs146794392/CM972912
17	*LAMB3*	c.958_1034dup (NM_000228.3)	p.Asn345Lysfs*77	Homozygous	Frameshift	Deleterious	Probably damaging	Disease causing	Pathogenic	Reported rs1553277702
18	*LAMB3*	c.1977-1G > A (NM_000228.3)	p.?	Homozygous	Missense	Deleterious	Probably damaging	Disease causing	Pathogenic	Reported rs786205451
**Epidermolysis bullosa simplex**										
19	*PLEC*	c.4552 C > T (NM_000445.5)	p.Gln1518*	Homozygous	Stop gained	Deleterious	Probably damaging	Disease causing	Pathogenic	Reported
										CM010392
20	*PLEC*	c.7144C > T (NM_000445.5)	p.Gln2382X*	Homozygous	Stop gained	Deleterious	Probably damaging	Disease causing	Likely pathogenic	Not reported
21	*PLEC*	c.7144C > T (NM_000445.5)	p.Gln2382X*	Homozygous	Stop gained	Deleterious	Probably damaging	Disease causing	Likely pathogenic	Not reported
22	*TGM5*	c.1335G > C (NM_004245.4)	p.Lys445Asn	Homozygous	Missense	Deleterious	Probably damaging	Disease causing	Pathogenic	Reported rs606231276/CM095542
23	*TGM5*	c.1335G > C (NM_004245.4)	p.Lys445Asn	Homozygous	Missense	Deleterious	Probably damaging	Disease causing	Pathogenic	Reported rs606231276/CM095542
24	*TGM5*	c.1335G > C (NM_004245.4)	p.Lys445Asn	Homozygous	Missense	Deleterious	Probably damaging	Disease causing	Pathogenic	Reported rs606231276/CM095542
25	*TGM5*	c.1138G > C (NM_004245.4)	p.Ala380Pro	Homozygous	Missense	Deleterious	Probably damaging	Disease causing	Pathogenic	Not reported
26	*DST*	c.3370C > T	p.Gln1124*	Homozygous	Stop gained	Deleterious	Probably damaging	Disease causing	Pathogenic	Reported
										CM103946
27	*DST*	c.16496C > G	p.Al5499Gly	Homozygous	Missense	Deleterious	Probably damaging	Disease causing	Likely pathogenic	Not reported
28	*KRT14*	c.1094_1095delGC	p.R365LfsX117	Heterozygous	Frameshift	Deleterious	Probably damaging	Disease causing	Likely pathogenic	Not reported

SIFT, <0.05 as deleterious; PolyPhen-2, the closer to 1 as probably damaging; MutationTaster, disease-causing is pathogenic variation; dbSNP#, The Single Nucleotide Polymorphism database; HGMD, The Human Gene Mutation Database.

## Discussion

DEB is a rare inherited EB caused by mutations involving the genes that encode type VII collagen leading to the separation of the sub-basal lamina (Brun et al., 2017). Recessive DEB has a wider array of severity and milder/localized form that has acral and nail involvement, similar to other forms of DEB. In particular, DEB patients have a significant risk of developing aggressive squamous cell carcinoma in chronic lesion sites (Mitsuhashi and Hashimoto 2003). The severe form is characterized by generalized blistering of the hands and feet, usually involving the acral surfaces, leading to pseudosyndactyly and flexural contractures that intensify with age (Bruckner-Tuderman 2010). Clinically, our cases do not differ much from what were described internationally. Although gastrointestinal complications are common in patients with EB, this was not the case in our patients since only one patient had pyloric atresia.

DEB was the most frequent subtype in the study population. Twelve patients with DEB were detected with 10 different homozygous variants in *COL7A1*. A total of 5 mutations are novel: 4 missense and 1 frameshift (Table 2). The other 5 reported variants are 4 missense mutations and 1 non-sense mutation that were previously reported (Horn and Tidman 2002b; Almaani et al., 2009). These results expand the spectrum of identified mutations implicated with DEB.

Six patients with JEB were among the study population. A total of 3 patients had novel mutations, and the other 3 patients had previously reported mutations. Two novel homozygous mutations were detected in 2 genes, *COL17A1* and *LAMB3*; 1 mutation with frameshift in *COL17A1*; and 1 frameshift in *LAMB3* gene (Table 2). In those with consanguineous history, homozygous mutations were identified. The reported mutations are 1 non-sense, 1 frameshift, and 1 missense in *LAMB3* genes, which were previously reported by Christiano et al. and Pulkkinen *et al.* (Christiano et al., 1996; Pulkinnen and Uitto, 1999).

EBS was diagnosed in 10 patients with 8 different mutations in 4 genes: *TGM5*, *PLEC*, *DST*, and *KRT14*. Four are pathogenic mutations that are not reported to date. The 4 novel mutations of EBS in 3 different genes are 1 missense in *TGM5*, 2 non-sense in *PLEC*, and 1 missense in *DST* (Table 2). The other 5 reported mutations are 2 missense in *TGM5*, one non-sense mutation involving *PLEC*, and one frameshift in *KRT14* gene that were previously reported (Fine and Mellerio 2009b; Fine et al., 2008; Mitsuhashi and Hashimoto 2003). *TGM5* mutations may belong to other disorders with skin fragility and not to classical EB (Has et al., 2020). Almost all cases (Table 2) were inherited in an autosomal recessive pattern harboring either homozygous or compound heterozygous allele variants, although the loss-of-function mutations might have resulted from missense or frameshift mutations through the mechanism of non-sense-mediated mRNA decay. Indeed, it seems likely that the presence of homozygosity in our Saudi patients conformed to a high fertility rate and consanguinity rate, which reached more than 50% in some areas (Scott et al., 2016).

It is worth mentioning that all newly reported variants in this study were evaluated for their impact on protein function and structure using in silico prediction tools such as Sorting Intolerant From Tolerant (SIFT), Polymorphism Phenotyping v2 (PolyPhen-2), and MutationTaster. However, the final protein function pathogenicity effect of these mutant variants and the association with the EB disease may require further functional verification.

### Study limitation

The study was conducted at a single tertiary care center at central SA, yet it is the first pillar to establish the Saudi EB genetic profile. Secondly, the retrospective design has made the study more subjective to missing some relevant data. However, the effects do not have any major impact on the findings. We propose therefore further collaboration between various centers from different parts of the region to have substantial effects on the sample size, including diversifying the patients’ backgrounds.

## Conclusion

The study elaborates and reports on the genetic profile and prevalence of EB in Saudi patients at a single tertiary healthcare center in central SA. Dystrophic EB was the highest reported subcategory among all the other EB classifications. *COL7A1* was the most common gene identified among the patients. Positive family history of consanguinity was evident as expected; further highlighting its role through education is needed among Saudi EB patients.

This study entails an impact on the future of identifying the genetic characteristics of Saudi EB patients, along with emphasizing on the need to launch an entity that would be responsible for directing the efforts of initiating a national center for EB.

## Data Availability

The raw data supporting the conclusions of this article will be made available by the authors, without undue reservation.

## Author’s Note

This manuscript is dedicated to Ahad Alharthi one of our interns who participated in this research and passed away on 27th July 2021. Despite her short professional life as a medical student and intern, she participated in many researches and was a leader in many skin awareness campaigns.
